# Discovery of PPAR Alpha Lipid Pathway Modulators That Do Not Bind Directly to the Receptor as Potential Anti-Cancer Compounds

**DOI:** 10.3390/ijms26020736

**Published:** 2025-01-16

**Authors:** Arwa Al Subait, Raghad H. Alghamdi, Rizwan Ali, Amani Alsharidah, Sarah Huwaizi, Reem A. Alkhodier, Aljawharah Saud Almogren, Barrak A. Alzomia, Ahmad Alaskar, Mohamed Boudjelal

**Affiliations:** 1 Medical Research Core Facility and Platforms (MRCFP)-Drug Discovery Platform, King Abdullah International Medical Research Center (KAIMRC), King Saud Bin Abdulaziz University for Health Sciences, Ministry of National Guard Health Affairs (MNGHA), Riyadh 11481, Saudi Arabia; alsubaitar@kaimrc.edu.sa (A.A.S.);; 2 Clinical Laboratory Sciences Department, College of Applied Medical Sciences, King Saud Bin Abdulaziz University for Health Sciences, Ministry of National Guard Health Affairs (MNGHA), Riyadh 11481, Saudi Arabia; 3 King Abdulaziz and His Companions Foundation for Giftedness and Creativity (MAWHIBA), Riyadh 11481, Saudi Arabia; 445009532@pnu.edu.sa; 4 College of Science, King Saud University, Riyadh 11459, Saudi Arabia; 441203711@student.ksu.edu.sa; 5 Department of Pharmaceutical Sciences, College of Pharmacy, King Saud Bin Abdulaziz University for Health Sciences, Ministry of National Guard Health Affairs (MNGHA), Riyadh 11481, Saudi Arabia

**Keywords:** PPAP alpha modulators, adipogenesis, anti-cancer, lipid metabolism

## Abstract

Peroxisome proliferator-activated receptors (PPARs) are considered good drug targets for breast cancer because of their involvement in fatty acid metabolism that induces cell proliferation. In this study, we used the KAIMRC1 breast cancer cell line. We showed that the PPARE-Luciferase reporter gets highly activated without adding any exogenous ligand when PPAR alpha is co-transfected, and the antagonist GW6471 can inhibit the activity. Using this reporter system, we screened 240 compounds representing kinase inhibitors, epigenetic modulators, and stem cell differentiators and identified compounds that inhibit the PPARα-activated PPARE-Luciferase reporter in the KAIMRC1 cell. We selected 11 compounds (five epigenetic modulators, two stem cell differentiators, and four kinase inhibitors) that inhibited the reporter by at least 40% compared to the controls (DMSO-treated cells). We tested them in a dose-dependent manner and measured the KAIMRC1 cell viability after 48 h. All 11 compounds induced the cell killing at different IC50 values. We selected two compounds, PHA665752 and NSC3852, to dissect how they kill KAIMRC1 cells compared to the antagonist GW6741. First, molecular docking and a TR-FRET PPARα binding assay showed that compared to GW6471, these two compounds could not bind to PPARα. This means they inhibit the PPARα pathway independently rather than binding to the receptor. We further confirmed that PHA665752 and NSC3852 induce cell killing depending on the level of PPARα expression, and as such, their potency for killing the SW620 colon cancer cell line that expresses the lowest level of PPARα was less potent than for the KAIMRC1 and MDA-MB-231 cell lines. Further, using an apoptosis array and fatty acid gene expression panel, we found that both compounds regulate the PPARα pathway by controlling the genes involved in the fatty acid oxidation process. Our findings suggest that these two compounds have opposite effects involving fatty acid oxidation in the KAIMRC1 breast cancer cell line. Although we do not fully understand their mechanism of action, our data provide new insights into the potential role of these compounds in targeting breast cancer cells.

## 1. Introduction

Nuclear receptors (NRs) represent one of the most important cellular transcription factors that regulate essential genes involved in different cell functions like differentiation, metabolism, detoxification, death, and survival. Several marketed drugs are nuclear receptor (NR) ligands for some diseases, including cancer, metabolic, and inflammatory disorders [1,2,3,4]. These ligands are either agonists or antagonists of the nuclear receptor and can induce cell apoptosis, differentiation, and metabolism [1,2]. A total of 48 nuclear receptors have been reported in humans that are regulated by endogenous ligands, and many are still named orphan receptors with their ligands to be discovered [5,6]. Nuclear receptors regulate transcription through binding as heterodimers, homodimers, or monomers to their response elements in the promoters of their target genes [2,7]. Among the nuclear receptors are the peroxisome proliferator-activated receptors (PPARs) that play an essential role in gene regulation through heterodimerization with the retinoid X receptor (RXR) and exist in three isoforms, namely, PPARα, PPARγ, and PPARβ/δ [8]. The receptors can bind both agonist and antagonist ligands. The former induces proper conformational changes to stabilize the PPARs and induce transcription. The resulting ternary complex binds to the peroxisomal proliferator response element (PPRE) on the promoter region of the target genes and drives gene transcription. However, the antagonist does the opposite, which does not lead to transcriptional complex activation [9]. The endogenous ligands of PPARs have been studied intensively and found to be either oxidized lipids, eicosanoid derivatives, or long-chain polyunsaturated fatty acids [10]. Among the nuclear receptors that have been paid much attention in breast cancer are the estrogen receptor alpha and beta (ERα, ERβ). The ERα is expressed in almost 70–80% of breast cancers, and that represents one of the best drug targets for non-metastatic conditions [11]. The function of ERβ is not clear in breast cancer, but it forms a heterodimer with ERα and induces distinct gene expression when compared to ERα or ERβ homodimers alone [12].

Alongside ERs, the PPARs are considered good drug targets for breast cancer. They are involved in fatty acid metabolism to induce cell proliferation. Inhibition of these receptors by long-chain n-3 polyunsaturated fatty acids can decrease the growth and metastasis of mammary tumors [13,14]. During fasting and glucose deprivation, PPARα expression is upregulated to activate the gene involved in fatty acid oxidation as an alternative source of fuel [15]. It allows the cell to be viable under metabolic stress. It has been remarkably found that the expression of PPARα and fatty acid oxidation are activated in diseased tissues such as melanoma, prostate cancer, and chronic lymphocytic leukemia (CLL) [16,17,18]. In PPARα knockout mice, many genes regulating fatty acid oxidation are downregulated. These mice are also protected from growing tumors [19]. PPARα has endogenous ligands that include oleoylethanolamide (OEA) [20], long-chain fatty acids, and leukotriene B4 (LTB4) [21]. Some companies have developed PPARα ligands that include the antagonists NXT629 [22], MK886 [23], and GW6471 [24] and the agonist fibrates WY-14,643 and GW7647 [25]. NXT629 has shown promising efficacy in reducing the tumor burden in mice models for ovarian cancer and melanoma, and GW6471 has also shown anti-tumor activity in vitro [15].

On the other hand, several studies have indicated that lipid metabolism is a key energy source for tumors to adapt to stressful environments [26]. As an example, the inhibition of fatty acid synthesis in tumors renders them more sensitive to chemotherapy and increases tumor differentiation for neuroblastoma. As a consequence, most of the tumor-associated signaling pathways like c-MYC, ERK, EGRFR and others get blocked [27].

In this study, we used the KAIMRC1 breast cancer cell line [28]. We showed that the PPARE-Luciferase reporter gets highly activated without any exogenous ligand when PPAR alpha is co-transfected, and can be inhibited by the antagonist GW6471. Using this system, we screened for compounds that inhibit the PPARα-activated PPARE-Luciferase reporter in KAIMRC1 cells and do not directly bind to PPAR alpha. The experimental details, the nature of the discovered compounds that inhibit PPARE-Luciferase, and their proliferation effect on cancer cell proliferation are fully detailed.

## 2. Results

### 2.1. PPAR Alpha Pathway Is Highly Active in KAIMRC1 Cells

**Figure 1:**
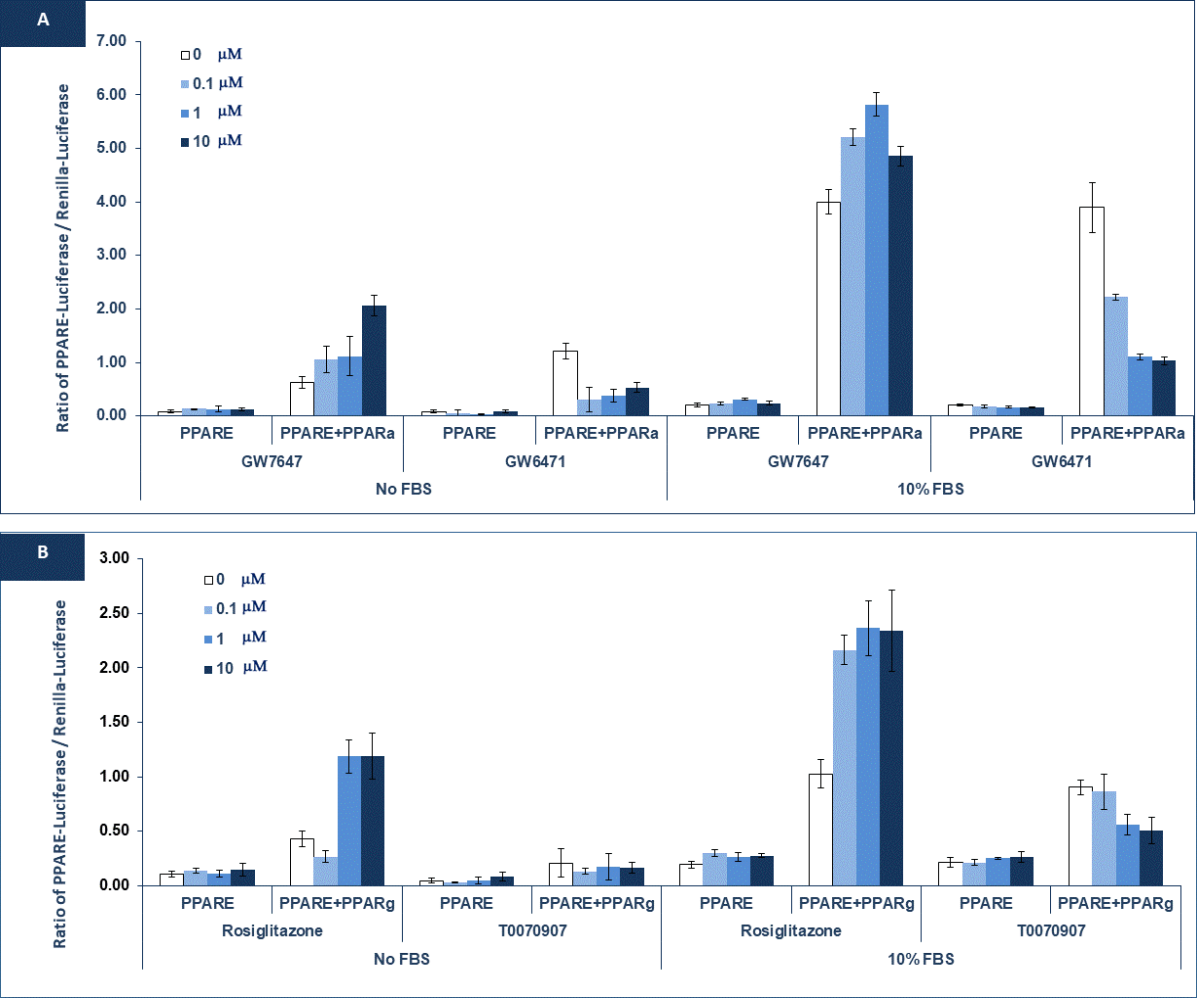
Activity of PPARE-Luciferase in KAIMRC1 cells: KAIMRC1 cells were transfected with PPARE-Luciferase (PPARE) alone, with PPAR alpha (PRα) (**A**) or PPAR gamma (**B**). As indicated, the cells were treated with PPAR alpha agonist (GW7647), antagonist (GW6471) or PPAR gamma agonist (Rosiglitazone), or antagonist (T0070907) in the absence (no FBS) or presence of 10% FBS. The ratio of PPARE-Luciferase/Renilla-Luciferase is blotted.

In our previous work, we studied the activity of nuclear receptors in the KAIMRC1 cell line [28] and found that their activity is distinct from that of the MCF7 and MDA-MB231 cell lines. These results highlight the importance of using KAIMRC1 as a novel breast cancer cell model in addition to other existing cell lines. In this study, we further examined PPAR alpha and gamma activity. As shown in Figure 1, PPARE-Luciferase was stimulated when the PPAR alpha and gamma receptors were co-transfected in the absence or presence of FBS. In the PPAR alpha co-transfection, adding the agonist (GW7647, Figure 1A) did not stimulate further the PPARE-Luciferase reporter. Treating cells with rosiglitazone in the PPAR gamma co-transfected cells remarkably increased the reporter activity in the absence and presence of FBS.

Remarkably, treating cells with the PPAR alpha antagonist GW6471 dramatically decreased the PPARE-Luciferase reporter (Figure 1A). Similarly, the PPAR gamma antagonist decreased the reporter with PPAR gamma, which was co-transfected (Figure 1B).

These data indicate that the PPARs’ pathway, especially PPAR alpha, is already activated in the KAIMRC1 cell line. The activation of the PPARE-Luciferase reporter in the absence of any exogenous ligand indicates that KAIMRC1 has an endogenous ligand that activates PPARs, especially PPAR alpha.

Our findings align perfectly with the existing literature, which states that cancer cells produce fatty acids as an extra fuel source for survival [29,30]. In this regard, the activation of the PPARE-Luciferase reporter, especially with PPAR alpha, suggests that KAIMRC1 cells generate fatty acids for their energy needs.

### 2.2. Discovery of PPAR Alpha Pathway Inhibitors in KAIMRC1 Cells

**Figure 2:**
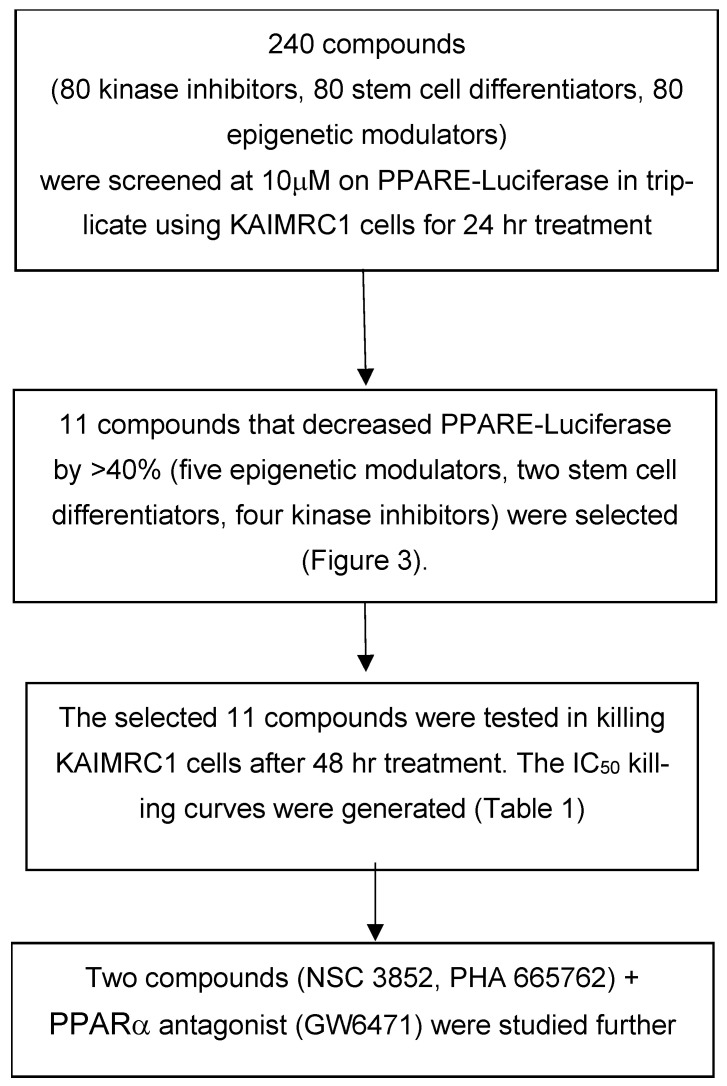
Schematic representation of screening cascade: KAIMRC1 cells transfected with PPARE-Luciferase and PPARα were treated with kinase inhibitors, epigenetic modulators, or stem cell differentiators. The identified compounds that inhibited the PPARE-Luciferase were characterized further as indicated.

We took advantage of this natural physiological activity to screen for compounds that could inhibit the PPARE-Luciferase reporter in KAIMRC1 cells activated by PPARα. Our rationale was that these compounds could also induce killing of KAIMRC1 cells and represent a good lead for further optimization as drugs. We were not interested in screening for PPAR alpha antagonists but rather for compounds that inhibit the PPARE-Luciferase reporter to identify compounds that inhibit the PPAR alpha pathway, not through antagonizing PPAR alpha. It is a novel approach that has never been pursued previously. Figure 2 represents the screening schematic to identify the compounds that inhibit the PPAR alpha pathway by inhibiting the PPARE-Luciferase reporter. A total of 240 compounds representing kinase inhibitors, epigenetic modulators, and stem cell differentiators, purchased from Tocris Bioscience (Minneapolis, MN, USA), were screened at a 10 µM final concentration for 24 h. In analyzing the data, we eliminated any compounds that inhibited or increased by more than 20% of renilla activity compared to DMSO as a control. The renilla reporter was used as the internal control, as compounds that affect the renilla by more than 20% either kill the cells or indirectly affect the luciferase and renilla reporter. In addition, we wanted to pick the best compounds that affected the PPARE-Luciferase reporters and did not kill the cells at this concentration after 24 h of incubation.

### 2.3. PPAR Alpha Pathway Inhibitors Induce KAIMRC1 Cells Killing

**Figure 3:**
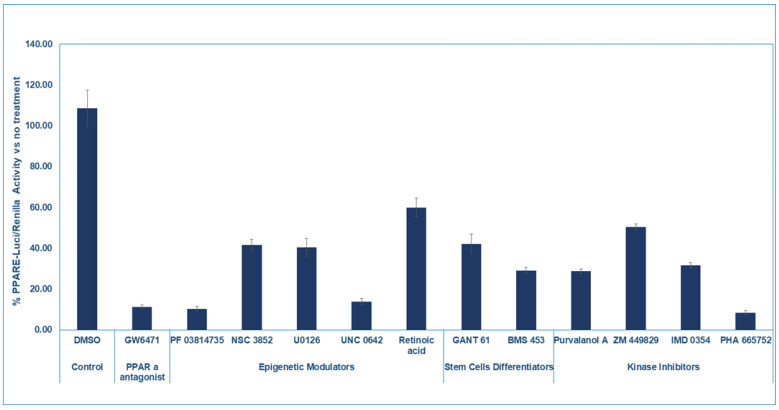
Inhibition of PPARE-Luciferase by selected compounds: the 11 compounds that inhibited by 40% the PPARE-Luciferase in the presence of PPAR alpha and did not affect the renilla or kill the KAIMRC1 cells were retested in the Luciferase reporter assay for confirmation after 24 h of treatment.

We selected 11 compounds (five epigenetic modulators, two stem cell differentiators, and four kinase inhibitors) that inhibited the reporter by at least 40% compared to the control wells (DMSO-treated cells). We repeated testing the compounds on the PPARE-Luciferase reporter for confirmation, as shown in Figure 3. In the same experiment, we tested the PPAPα antagonist GW6471 to demonstrate that PPARE-Luciferase activity can be reduced by direct inhibition of PPAR alpha. Also, we confirmed that these compounds did not kill the cells after 24 h of treatment. The 11 compounds represent different types of compounds modulating distinct targets that include aurora kinase A and B, histone deacetylase, MEK1 and 2, histone lysine methyltransferase, retinoic acid receptors, Cdk, JAK3, IκB, and c-Met, as shown in Table 1.

Based on the published data indicating that PPAR alpha antagonists induce cell death both in vitro and in vivo in animal models that include GW6471 [24,31,32], we hypothesized that our 11 compounds, which inhibit the PPAR alpha pathway but not PPAR alpha directly, would also induce the killing of KAIMRC1 cells. In this regard, we dose-dependently tested these compounds for 48 h and measured the KAIMRC1 cell viability after 48 h. As shown in Table 1, all the 11 compounds induced cell killing at different IC_50_ values. The most potent compound that killed the cells at an IC_50_ of 0.2967 was NSC3852, which is a pan-histone deacetylase inhibitor [33], followed by IM 0354 and UN 0642, which killed the KAIMRC1 at IC_50_ values of 1.682 and 1.879, respectively. These two compounds are inhibitors of IκB kinase-2 (IKK-2, IKK-β) [34] and G9a and GLP histone lysine methyltransferase inhibitor [35], respectively. The fourth and fifth compounds that killed the cells at IC_50_ values of 2.483 and 3.244 were PHA665752, which is a potent, selective, and ATP-competitive inhibitor of c-Met kinase [36], and PF 03814735, which is an aurora kinase A and B inhibitor [37]. The remaining compounds killed the cells at concentrations ranging from IC_50_ values of 8 to 122 µM. The control compounds, Doxorubicin, Mitoxantrone, and GW6471, killed the cells at IC_50_ values of 0.07579, 0.01359, and 0.05605 µM, respectively.

**Table 1:** IC_50_ killing curve of selected compounds: 11 identified compounds (epigenetic modulators, stem cell differentiators, kinase inhibitors) together with GW6471, Doxorubicin, and Mitoxantrone as controls were tested, killing KAIMRC1 cells at 11 points, concentration with the highest concentration of 50 µM, and dilution factor of 3. Titer Glo measured the killing after 48 h of treatment.

Compound		IC_50_ (µM)	R^2^	Target/Reference
**Control**	Doxorubicin	0.07579	0.9522	Inhibit DNA synthesis by intercalation and inhibit topoisomerase II [38,39]
	Mitoxantrone	0.01359	0.9695	
	GW6471	0.05605	0.9368	PPAR alpha antagonist [24]
**Epigenetic Modulators**	PF 03814735	3.336	0.9024	Aurora kinase A and B inhibitor [37]
	NSC3852	0.2967	0.9479	Pan-histone deacetylase inhibitor [33]
	U0126	118.4	0.9360	Potent selective inhibitor of MEK1 and 2 [40]
	UNC 0642	1.879	0.9918	G9a and GLP histone lysine methyltransferase inhibitor [35]
	Retinoic acid	52.37	0.9338	Activator of retinoic acid receptors [41]
**Stem Cell Differentiators**	GANT 61	7.242	0.957	Transcription factors [42]
	BMS 453	297.6	0.9450	Synthetic retinoid: RARβ agonist; RARα and RARγ antagonist [43]
**Kinase Inhibitors**	Purvalanol A	17.95	0.7617	Cdk inhibitor; potently inhibits cdk1, cdk2, and cdk5 [44]
	ZM 449829	8.332	0.9006	Potent selective inhibitor of Janus tyrosine kinase 3 (JAK3) [45]
	IMD 0354	1.682	0.9698	Inhibitor of IκB kinase-2 (IKK-2, IKK-β) [34]
	PHA665752	2.483	0.9816	Potent, selective, and ATP-competitive inhibitor of c-Met kinase [36]

### 2.4. Characterize the Killing Mechanism of PPAR Alpha Pathway Inhibitors in KAIMRC1 Cells

To understand the pathway by which the newly discovered compounds inhibited the PPAR alpha pathway in KAIMRC1 cells and induced its killing after 48 h of treatment, we chose two compounds: PHA665752, which is a potent, selective, and ATP-competitive inhibitor of c-Met kinase [36], and NSC3852, which is a pan-histone deacetylase inhibitor [33]. Then, we tested their ability to bind PPAR alpha and their PPAR alpha cell killing dependency and analyzed their effects on the lipid and apoptosis pathway.

#### 2.4.1. PHA665752 and NSC3852 Do Not Bind PPAR Alpha

We carried out molecular docking calculations to exclude the possibility that the newly discovered inhibitors bind to PPARα and modulate its function. Only one X-ray crystal structure of PPARα is available in the inactive antagonist-bound state (PDB ID: 1KKQ) at a 3 Å resolution [24]. It is a ternary complex of the receptor GW6471 antagonist and SMRT co-repressor peptide. The PPARα LBD comprises 13 α-helices and four β-strands [24]. Hydrogen bonding to Tyr 464 on the activation helix 12 (H12) has been found to be essential for agonist activity [46], and the loss of this interaction gives rise to the antagonist GW6471 [24]. The presence of GW6471 pushes H12, which in turn renders the receptor unable to assume an active structured conformation [24]. GW6471 also contributes to receptor inactivation by inducing an LBD conformation favorable for efficient interactions with the SMRT co-repressor, improving the binding of the co-repressor to the receptor [24]. GW6471 has been found to adopt a U-shape conformation in the LBD of PPARα while wrapping around Cys 276 of H3 [24]. For antagonist activity, a compound must be in proper orientation in the binding pocket to push H12 out so it does not assume an active conformation [24]. In another study, interactions with Tyr 314 have also been found to be important for antagonist activity [47].

**Figure 4:**
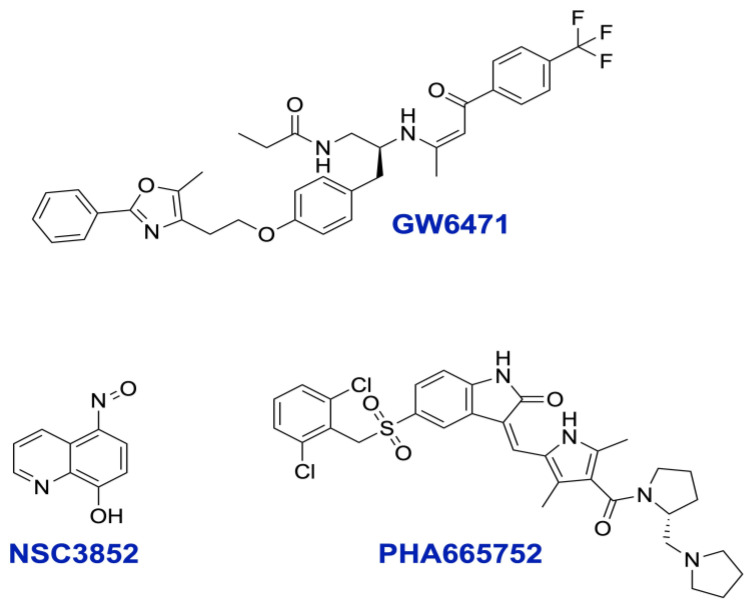
Structures of GW6471, NSC3852, and PHA665752.

**Figure 5:**
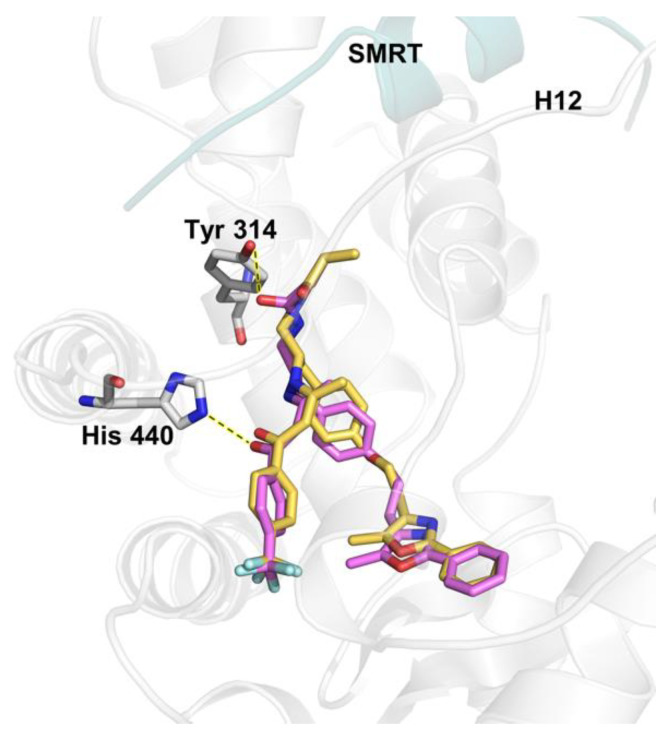
Overlay of the docked pose of GW6471 (magenta sticks) and the X-ray crystal structure pose (yellow sticks). The SMRT co-repressor is shown as a teal carton, residues as platinum sticks, and hydrogen bonds as yellow dashed lines.

The co-crystallized antagonist GW6471 (Figure 4) was docked back into the PPARα X-ray crystal structure (PDB ID: 1KKQ) [24] to ensure Autodock Vina’s [48,49] ability to predict the correct binding pose. The docking of GW6471 had a U-shape conformation with a low RMSD value of 0.961, validating Autodock Vina’s [48,49] ability to reproduce the experimental binding mode accurately. The docked pose of GW6471 showed hydrogen bonds with His 440 and Tyr 314 (Figure 5) and a docking score of −11.1 kcal/mol.

**Figure 6:**
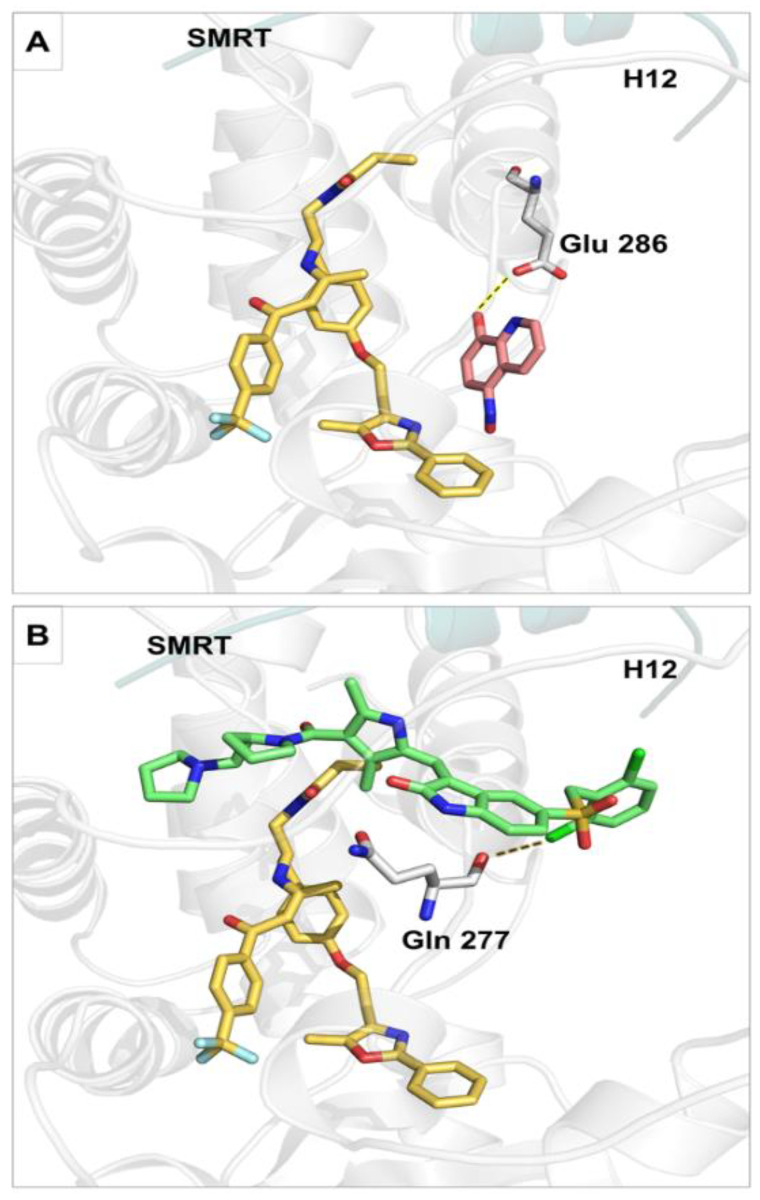
The docked poses of (**A**) NSC3852 (pink sticks) and (**B**) PHA665752 (green sticks). The X-ray crystal structure pose of GW6471 is shown as yellow sticks, the SMRT co-repressor as a teal carton, residues as platinum sticks, hydrogen bonds as yellow dashed lines, and halogen bonds as gold dashed lines.

The compounds NSC3852 and PHA665752 (Figure 5) were also docked into the LBD of PPARα to examine their potential to act as antagonists. NSC3852 had a docking score of −6.8 kcal/mol, while PHA665752 had a docking score of −8.9 kcal/mol, indicating that GW6471 may bind better to the receptor than these two compounds. NSC3852 was bound in a different part of the binding pocket than GW6471, away from Tyr 314, and not in a position that can push H12 out to give an inactive conformation. NSC3852 made a hydrogen bond with Glu 286 (Figure 6A). PHA665752 made a halogen bond with Gln 277 and had a different pose with different orientations of head groups and tail groups compared to GW6471 (Figure 6B). The lack of proper compound orientation in the correct part of the binding pocket and the absence of interactions with important residues such as Tyr 314 suggest that compounds NSC3852 and PHA665752 are not likely to function as PPARα antagonists.

**Figure 7:**
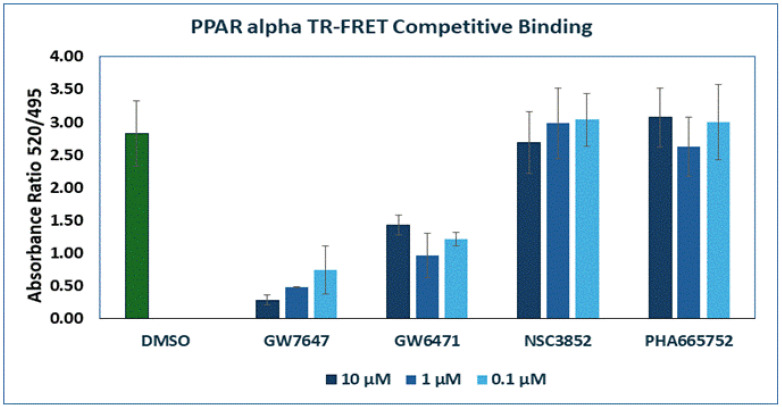
TR-FRET-based competitive binding assay for PPAR alphawas conducted for one hour for the compounds GW7647, GW6471, NSC3852, and PHA665752 at three concentrations (0.1, 1, and 10 µM). GW7647 was used as a positive (agonist) ligand control for PPAR α. GW6471 was used as an antagonist for PPAR alpha, while DMSO was used as a negative control.

To confirm that NSC3852 and PHA665752 do not bind to PPAR alpha, we carried out a TR-FRET-based competitive binding assay as described in the Section 3. As shown in Figure 7, the PPAR alpha agonist GW7647 competed with the PPAR alpha TR-FRET probe dose-dependently, demonstrating that GW7647 directly binds to PPAR alpha. The PPAR alpha antagonist GW6471 also competed with the probe and bound to PPAR alpha. However, the compounds NSC3852 and PHA665752 showed no competition with the probe and binding at all tested concentrations. These data support our modeling study that found that the two compounds do not bind to PPAR alpha.

To better understand the molecular mechanism of how NSC3852 and PHA665752 could inhibit the PPAR alpha pathway and induce cell killing, we modeled them in their possible targets.

Since NSC3852 is a pan-histone deacetylase (HDAC) inhibitor [33,50] and PHA665752 is a hepatocyte growth factor receptor (c-Met kinase) inhibitor [36], they were docked into their corresponding targets to gain insights into their binding. Targets with available X-ray crystal structures were used. NSC3852 was docked into SIRT1, HDAC1, HDAC2, HDAC4, HDAC6, HDAC7, and HDAC8, while PHA665752 was docked into c-Met kinase (Table 2). The co-crystallized inhibitor of each target was docked for validation. Most of the resultant RMSD values were within acceptable limits, the highest being for a peptide inhibitor of HDAC1. This could be due to the high flexibility of peptides. In most cases, the docking scores of the two compounds were higher than those of the co-crystallized inhibitors. NSC3852 and PHA665752 had different or fewer interactions with the targets compared to the co-crystallized inhibitors. Knowing that NSC3852 is a pan-histone deacetylase inhibitor [33,50] and PHA665752 is a c-Met kinase inhibitor [36], our docking studies provide insights into their binding. These insights pave the way for further studies to investigate the compounds’ inhibition of the targets and to determine whether other amino acids are important for target inhibition.

#### 2.4.2. NSC3852 and PHA665752 Cell Killing Dependency on PPAR Alpha Overexpression

**Figure 8:**
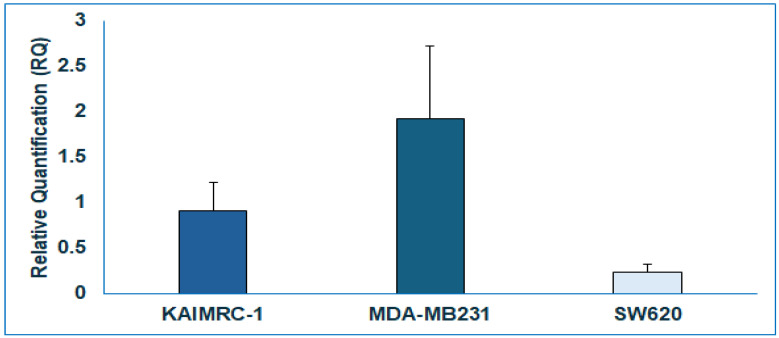
PPARα expression in different cancer cell lines. Real-time quantitative PCR showing the relative quantification (RQ) of PPARα in KAIMRC1, MDA-MB231, and SW620 cells. The cells were grown for 48 h for RNA isolation, and cDNA was synthesized using gene-specific primers. Relative quantification values are means (bars) ± and standard deviations (error bars) from three biological replicates.

As we demonstrated that NSC3852 and PHA665752 inhibited PPARα luciferase activity and induced cell death without binding to PPARα, we hypothesized that their molecular mechanism for cell killing does not involve directly antagonizing PPARα but rather inhibits the pathway modulated by PPARα. To test this hypothesis, we first compared the potency of NSC3852 and PHA665752 in destroying cells with varying levels of PPARα expression. According to The Human Protein Atlas database [59], the colorectal cancer cell line SW620 exhibits the lowest PPARα expression. We further validated this data by quantifying the mRNA expression in this cell line using QPCR, comparing it to the KAIMRC1 cell. As shown in Figure 8, PPARα mRNA expression was nearly 80% lower in the SW620 cell line (0.299 ± 0.185) compared to the KAIMRC1 cell (1.133 ± 0.702), while the MDA231 cell had (2.489 ± 1.370).

**Figure 9:**
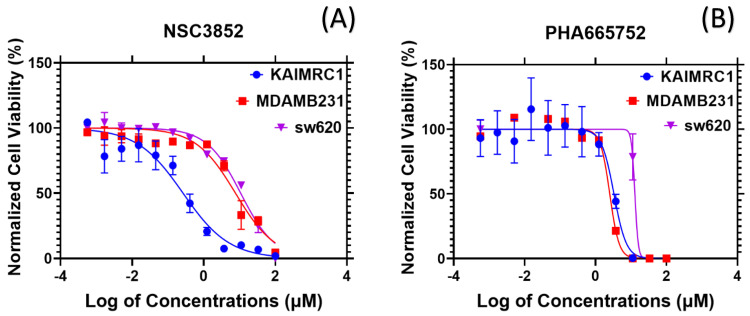
Dose-response curve for the half-maximal inhibitory concentration IC_50_ (μM) of (**A**) NSC3852 and (**B**) PHA665752 against KAIMRC1, MDAMB231, and SW620 cell lines. The X-axis is the log of concentrations in µM, and the Y-axis is normalized cell viability in percentages.

We then tested the ability of NSC3852 and PHA665752 to kill SW620 cells compared to KAIMRC1 and MDA-231 cells. Figure 9 shows the killer curve for PHA665752 and NSC3852 against KAIMRC1, MDAMB231, and SW620 cell lines, while Table 3 showcases the IC_50_ values for PHA665752 and NSC3852. Both compounds demonstrated high potency against KAIMRC1 (0.26 and 3.3 µM, respectively) and comparatively low potency against SW620. The colorectal cancer cell line SW620, which had diminished expression of PPARα, showed high IC_50_ values (10.8 and 12.8 µM, respectively), confirming the involvement of PPARα expression in cell killing. MDA-MB-231, which had a relatively high expression of PPARα, showed more killing with compound PHA665752 compared to NSC3852. These results reflect that while PPARα seems to modulate killing in the cells, it does so at different rates across different cell lines. They also indicate that other factors are involved alongside PPARα modulation.

These data support our hypothesis that NSC3852 and PHA665752 induce cell killing depending on the activation presence of PPARα and the high activity of its pathway in cells.

**Table 3:** IC_50_ and R^2^ values for PHA665752 and NSC3852.

	KAIMRC1		MDAMB231		SW620	
	IC_50_	R^2^	IC_50_	R^2^	IC_50_	R^2^
**NSC3852**	0.26	0.93	7.7	0.91	10.82	0.97
**PHA665752**	3.3	0.94	2.5	0.99	~12.8	0.97

#### 2.4.3. Effect of PPAR Alpha Pathway Inhibitors on Stress and Apoptosis Pathways

We analyzed stress and apoptosis pathways after treatment with NSC3852 and PHA665752. As a control, we used the GW6471 compound.

**Figure 10:**
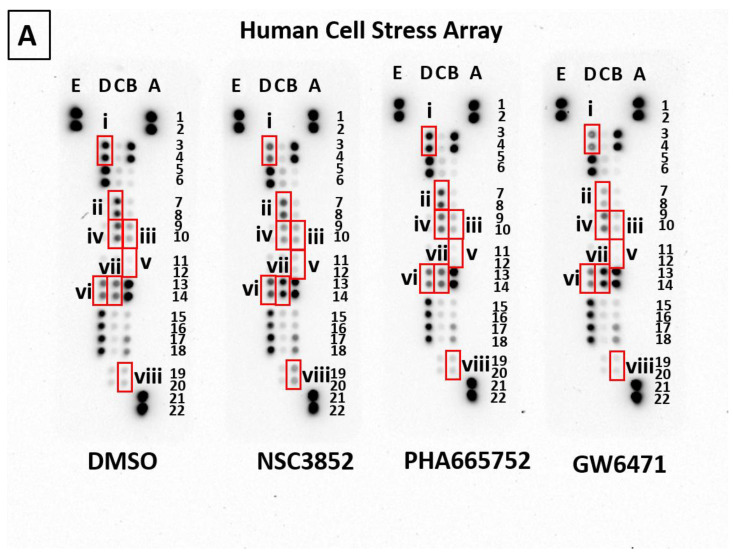

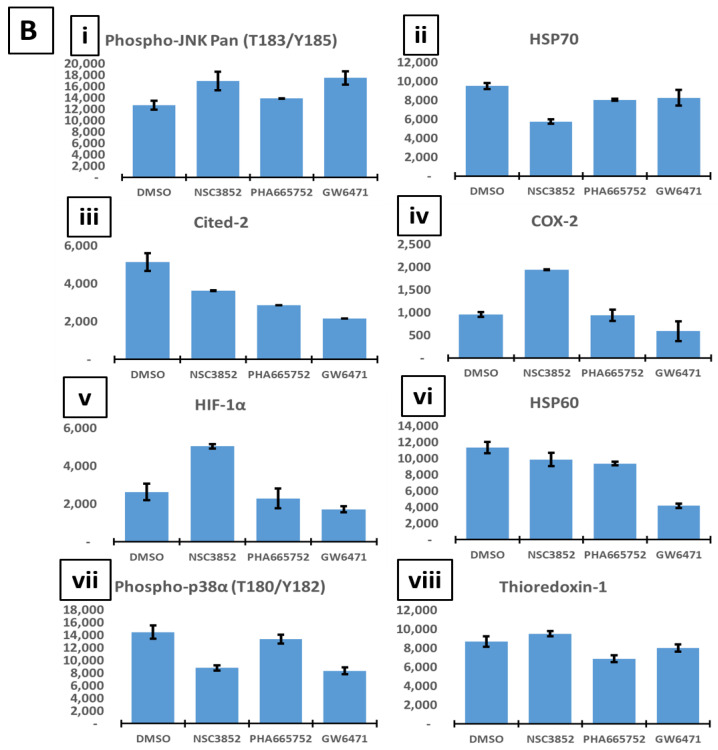
Effect of PPAR alpha pathway inhibitors on KAIMRC1 cell stress pathway: (**A**) post-treatment multiplex protein profiling of KAIMRC1 cells. The human cell stress array was utilized to detect the expression of key proteins involved in the PPARα inhibition pathway. (**B**) Graphical representation of selected analytes. The X-axis shows compounds, and the y-axis denotes the mean pixel density.

Figure 10A,B show the expression of HSP60, HSP70, CITED-2, and Phospho-p38α after treating KAIMRC1 cells with NSC3852, PHA665752, and the antagonist GW6471. HSP60 and HSP70 are molecular chaperones involved in the folding, translocation, and assembly of proteins. They play a crucial role in maintaining mitochondrial proteostasis [60]. CITED-2 and p-P38α are involved in transcription regulation. CITED-2 regulates/attenuates primary breast tumor growth, likely by influencing tumor vasculature via TGF-beta-dependent regulation of VEGFA [61]. CITED-2 has been implicated as a negative regulator of HIF 1α [62]. However, more comprehensive studies are needed to validate it [63]. Interestingly, CITED-2 is a direct effector of PPARɣ [64] and stimulates PPARα transcriptional activity. In contrast, p-P38α is a member of the MAP kinase family and is involved in cell proliferation, differentiation, and transcription regulation [65].

Notably, treatment with NSC3852 showed an increase in CO_2_ and HIF-1α expression. HIF-1α is an oxygen-dependent transcriptional activator crucial in tumor angiogenesis and mammalian development. It is known to regulate fatty acid synthesis and lipid storage. Interestingly, compound NSC3852 showed a 2-fold increase in the expression of HIF1α, indicating that it aids in increasing fatty acid synthesis. HIF-1α is a key regulator of cancer cell metabolism under hypoxic conditions [66]. Synthesis of the HIF-1α protein can be induced by various growth factors and cytokines, including insulin, insulin-like growth factors, and PDGF [67]. Meanwhile, Stamatakis et al. (2015) [68] reported that stable COX-2 overexpression in carcinoma cell lines (HT-29, HCT116, and Caco2) significantly impacts gene transcription. COX-2 is directly regulated by HIF-1α, and our results confirmed this when comparing their expression levels in Figure 10B(v,viii).

**Figure 11:**
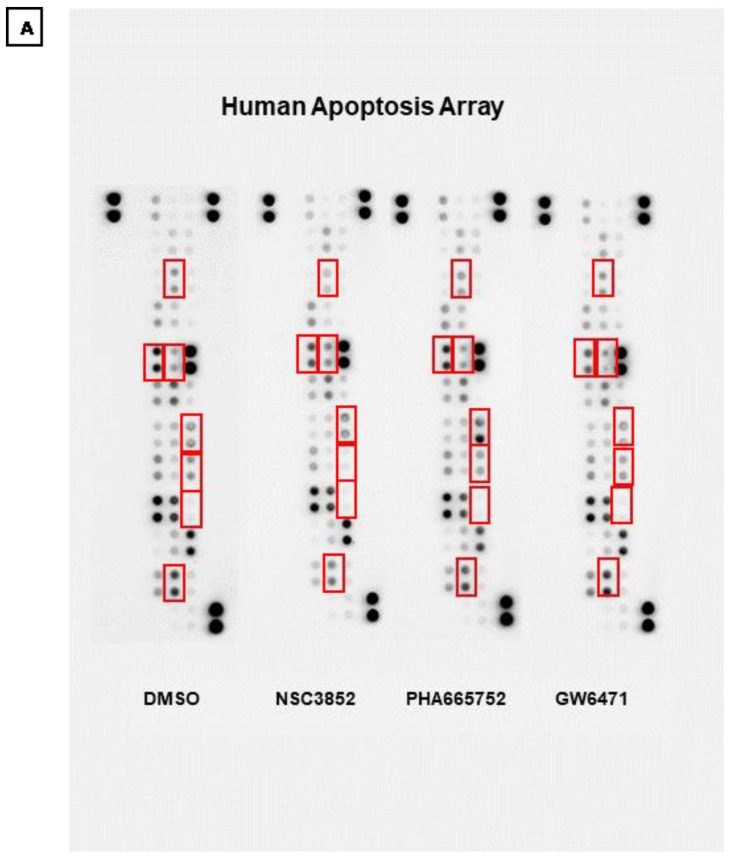

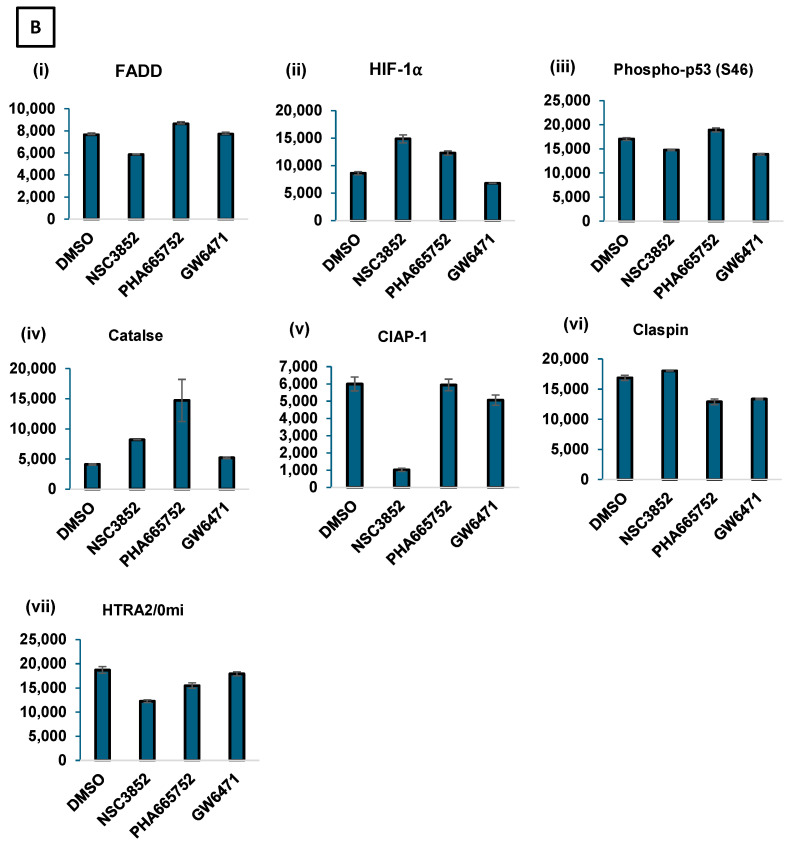
Profiling apoptosis proteins in treated KAIMRC1 cells. (**A**) Arrays were incubated with 400 µg of KAIMRC1 cell lysates treated with DMSO, NSC3852, PHA66575, and GW6471. The human apoptosis array detects multiple apoptosis-related proteins in treated KAIMRC1 cell lysates. Each protein was spotted in duplicate. The pairs of dots in each corner are the positive controls. (**B**) Graphical representation of selected lysates. The X-axis shows PPARA alpha modulators and the Y-axis denotes averaged pixel density.

To explore the molecular basis of the apoptotic effect of PPARα modulators (NSC3852 and PHA665752) in comparison to the PPARα antagonist (GW6471) in KAIMRC1 breast cancer cells, the human apoptosis array was used to detect the expression of apoptotic proteins. As shown in Figure 11, KAIMRC1 cell lysates treated with PPARα modulators showed variable apoptosis protein expression. The expression of Catalase, FADD, and phospho-p53(S46) was increased in the KAIMRC1 cells treated with PHA665752. Catalase is a heme enzyme that catalyzes the conversion of hydrogen peroxide into hydrogen and oxygen. Increased catalase indicates increased fatty acid oxidation. It is a marker that the cells are going through stress and death [69].

Furthermore, the Fas-associated Death Domain (FADD) is an adaptor protein inducing apoptosis. In cancer, loss of FADD inhibits apoptosis and induces tumor cell survival. Therefore, it holds significant promise as a therapeutic option in treating cancer [70]. In addition, the p53 tumor suppressor protein induces apoptosis in cells with DNA damage. Phosphorylation of p53 at serine 46 (S46) guides cells to undergo apoptosis rather than cell cycle arrest [71]. Taken together, our results suggest that PHA665752 may activate cell death of cancer cells by inhibiting the PPAR alpha pathway.

Notably, the KAIMRC1 cells treated with NSC3852 showed a decrease in cIAP-1 and HTRA2/0mi expression. cIAP-1 inhibits apoptosis by inducing constitutive RIP1 ubiquitination in cancer cells [72]. Interestingly, when the KAIMRC1 cells were treated with NSC3852, cIAP-1 expression was downregulated compared to the cells treated with DMSO, PHA665752, or DMSO. Furthermore, HTRA2/0mi is a mitochondrial serine protease released into the cytosol during apoptosis to antagonize inhibitors of apoptosis (IAPs) and to contribute to caspase-independent cell death [73]. The KAIMRC1 cells treated with NSC3852 showed an increased expression level in HTRA2/0mi compared to cIAP-1, which may support the antagonizing effect theory.

However, the KAIMRC1 cells treated with NSC3852 significantly increased HIF-1 alpha expression. HIF-1 alpha is an oxygen-dependent transcriptional activator that is crucial for tumor angiogenesis. Additionally, it is well established that HIF-1 alpha regulates fatty acid synthesis and lipid storage, which is another apoptosis marker [74]. Moreover, Claspin, another apoptotic protein, was elevated in the KAIMRC1 cells treated with NSC3852. Claspin is a human checkpoint protein degraded during apoptosis and stabilized during cell survival. Our results suggest that NSC3852 may promote cell survival under certain conditions.

#### 2.4.4. Effect of PPAR Alpha Pathway on Fatty Acid Metabolism in KAIMRC1 Cells

To investigate the biological functions of the PPARα modulators NSC3852 and PHA665752, a fatty acid metabolism PCR array was applied to identify the potential target genes that were affected upon the treatment of the KAIMRC1 cells with PPARα modulators (NSC3852 and PHA665752). The differential mRNA expression levels of 84 genes involved in fatty acid metabolism were assessed. The assessed genes were involved in different signaling pathways, including (a) fatty acid transport, biosynthesis, and regulation, (b) ketogenesis and ketone body metabolism, (c) triglycerol metabolism, and (d) fatty acid metabolism.

**Figure 12:**
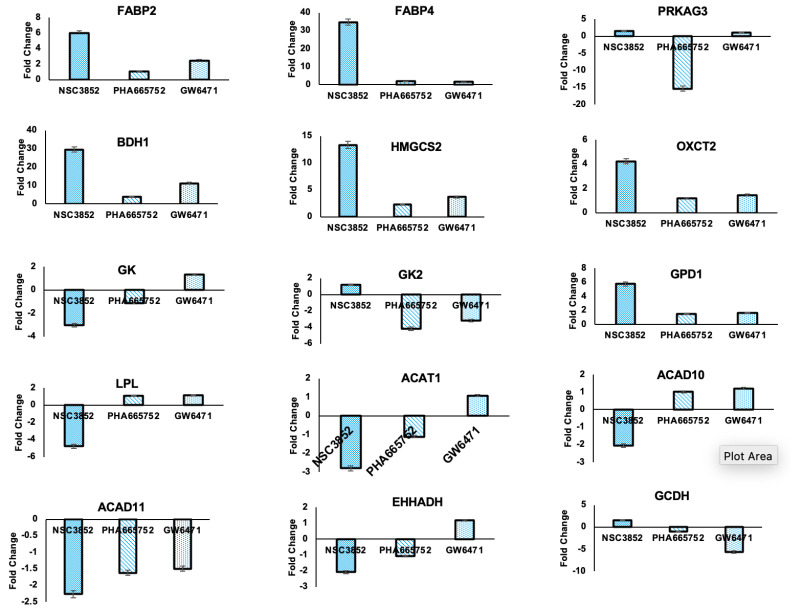

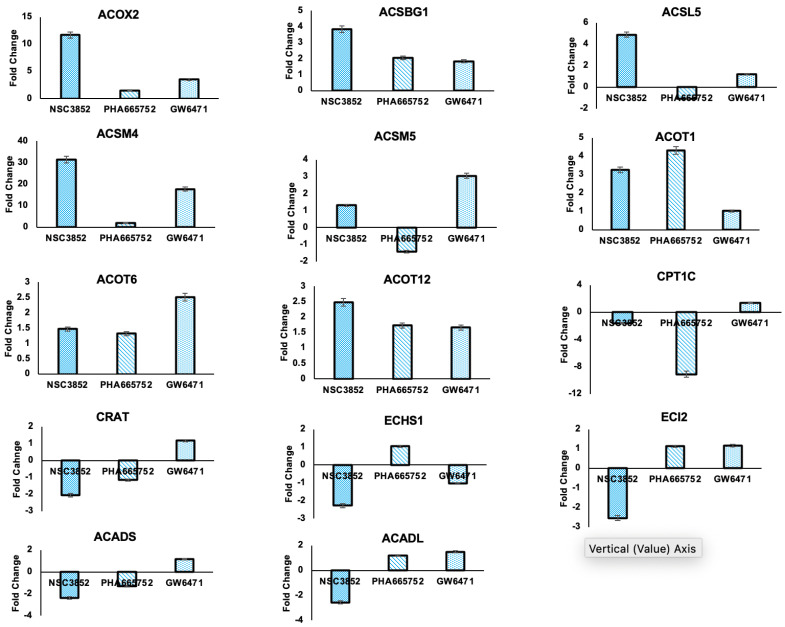
Effect of PPAR alpha pathway inhibitor of expression of fatty acid metabolism genes. Gene expression of human fatty acid metabolism genes in KAIMRC1 cells treated with PPARα modulators (NSC3852 and PHA665752) in comparison to the PPARα antagonist (GW6471).

As shown in Figure 12, in the KAIMRC1 cell lysates treated with PPARα modulators, 29 out of the 84 tested genes showed changes in their expression compared to the control group. The KAIMRC1 cells treated with NSC3852 showed increased fatty acid binding proteins FABP2 and FABP4 with a 6.02- and 34.82-fold change, respectively. FABP4 overexpression is a tumor-promoting molecule in most cancer types, suggesting that NSC3852 may inhibit histone deacetylation and promote fatty acid transportation via FABP2 and/or FABP4. On the other hand, an energy sensor protein, protein kinase AMP-activated non-catalytic subunit gamma 3 (PRKAG3), which plays a key role in regulating cellular energy, had downregulated expression in the KAIMRC1 cells treated with PHA665752, with a −15.33-fold change compared to the control cells. To further investigate the role of PPARα modulators, we analyzed the expression levels of six key genes involved in ketogenesis and ketone body metabolism in the KAIMRC1 cells treated with NSC3852 and PHA665752. Out of six genes, three genes were significantly upregulated in the KAIMRC1 cells treated with NSC3852. BDH1, an enzyme involved in ketogenesis and ketolysis, was the most upregulated (~30 fold). The other two upregulated genes were HMGCS2, a mitochondrial enzyme that modulates ketogenesis [75], and OXCT2, an essential enzyme in ketone body catabolism, with 13.4- and 4.2-fold changes, respectively.

Regarding triglycerol metabolism, in the KAIMRC1 cell lysates treated with NSC3852, the GPD1 gene was upregulated (5.7-fold), and LPL and GK were downregulated (−4.7-fold and −3-fold changes, respectively). On the other hand, the cell lysates treated with PHA665752, GK, and GK2 were downregulated (−1.1-fold and −4.1-fold changes, respectively). Moreover, in the KAIMRC1 cells treated with NSC3852, six genes involved in fatty acid metabolism were significantly upregulated. ACSM4 was the most upregulated gene (31.5-fold), followed by ACOX2 (11.7-fold), ACSL5 (4.9-fold), ACSBG1 (3.8-fold), ACOT1 (3.3-fold), and ACOT12 (2.5-fold). It has been reported that ACOX2 could promote cell proliferation of ER^+^ breast cancer cells [76]. In contrast, the KAIMRC1 cells treated with PHA665752 showed a significant decrease in carnitine palmitoyltransferase 1 C (CPT1C), with a −9.12-fold change compared to the control cells. CPT1C is a well-known fatty acid oxidation gene frequently upregulated in cancer cells [77]. In addition, long-chain acyl-CoA synthetase-5 (ACSL5) was downregulated in the cells treated with PHA665752 (−1.07-fold) but not in the cells treated with NSC3852. A previous study reported that long-chain acyl-CoA synthetases have essential roles in fatty acid activation in cancer cells [78].

## 3. Materials and Methods

### 3.1. Biological and Chemical Reagents

General biological reagents listed elsewhere (e.g., buffers) were purchased from Sigma-Aldrich and Roth (Taufkirchen, Germany) and were of the highest purity. The kinase, stem cell, epigenetic drug set, and other compounds were purchased from Tocris Bioscience (Minneapolis, MN, USA).

### 3.2. Cell Culture

Human breast cancer cells KAIMRC1 [28] and MDA-MB-231 (ATCC, HTB-26), along with colorectal cancer cells SW620 (ATCC, CCL-227), were maintained in Dulbecco’s Modified Eagle Medium (DMEM) supplemented with 10% fetal bovine serum (FBS), 50 units/mL penicillin, and 50 μg/mL streptomycin (Gibco, Gaithersburg, MD, USA), along with 2 mM L-glutamine (Gibco) at 37 °C in a humidified 5% CO_2_ atmosphere. All the proliferation and reporter assays were performed in the same media with the specified % FBS.

### 3.3. Cell Transfection and Luciferase Reporter Assays

In a six-well plate, 1–2 × 10^6^ KAIMRC1 cells in 2 mL complete media were seeded in each well. The cells were left to attach to the plate for 2 h before the transfection. The transfection mix was prepared as follows: 2.5 μg PPARE(3)-tk-Luciferase alone, or with 0.5 μg hPPARa pcDNA3.1-hPPARalpha, or with pSG5-hPPARgamma plasmids were mixed in 1.5 mL Eppendorf using 100 μL Opti-MEM I Reduced Serum Media (Thermo Fisher Scientific, Bartlesville, OK, USA). In all conditions for renilla measurement, 0.5 μg pRL-CMV Vector plasmid was co-transfected. 12 μL FuGENE^®^ 6 Transfection Reagent (Promega, Madison, WI, USA) was added to the transfection tube drop-wise. The plasmids and Fugene transfection mix were incubated at room temperature for 8–10 min. Then, each transfection mix was added to each cell culture well separately, containing 1–2 million cells/2 mL media. The transfection plates were incubated in a CO_2_ incubator overnight, and the next day, the media were replaced with fresh ones containing either 0% (serum-free) or 10% FBS as indicated. The cells were cultured for 24 h before seeding on compound plates. The reporter luciferase assays were performed using the Dual-Glo^®^ Luciferase Assay System (Promega), as detailed in our previous paper [28].

### 3.4. Cell Proliferation Assays

To obtain the IC_50_, the KAIMRC1, MDA-MB-231, and SW6420 cells were treated with a selected panel of commercially available compounds. Briefly, the cells were seeded at 10,000 cells/well into 96-well plates and incubated in a 5% CO_2_ incubator overnight. Later, the cells were treated with compounds in serial dilution followed by incubation for 48 h. CellTitre-GLo^®^ (Promega, USA) assays were performed after 48 h, and luminescence was measured using the Perkin Elmer Envision Instrument. The luminescence readings were normalized and expressed as a relative percentage. The data were analyzed using GraphPad Prism 8 software, and the half-maximal inhibitory concentration (IC_50_) was determined. Error bars denote standard deviation (SD).

### 3.5. Human Proteome Profiler Array

Briefly, the KAIMRC1 cells in six-well plates were treated with the vehicles GW6471, NSC3852, and PHA665752 for 48 h, and the cell lysates were isolated. Later, we used a Human Cell Stress Array Kit (Catalog # ARY018, R&D Systems, Minneapolis, MN, USA) and a Human Apoptosis Array Kit (Catalog# ARY009, R&D Systems, Minneapolis, MN, USA) to analyze the expression profile of the proteins that might be involved in the inhibition of PPARα. It contains 22 antibodies printed in duplicate. The kits were used according to the manufacturer’s protocol.

### 3.6. RNA Extraction and cDNA Synthesis

The KAIMRC1, MDA-MB-231, and SW620 cell pellets were lysed and homogenized following the manufacturer’s protocol. The total RNA was isolated using an RNeasy Mini extraction kit (Qiagen, Hilden, Germany, Cat # 74104). In brief, 70% ethanol was added to the cell lysate. Then, the sample was applied to a mini spin column to allow selective RNA binding to the membrane. Next, the column was washed three times to remove any contaminants. Finally, RNA was eluted using RNAse-free water, and our samples’ purity and concentration were determined using a Nanodrop spectrophotometer. First-strand cDNA was synthesized using an RT^2^ first-strand kit (Qiagen, Cat# 330401), following the manufacturer’s protocol.

### 3.7. RT^2^ Profiler PCR Arrays

PCR amplification was performed using an RT^2^ SYBR Green Fluor PCR mix (Qiagen, Cat. # 330510). From each sample, 20 µL of the product was used to run the RT^2^ Profiler PCR array (Qiagen, cat# 330231, GeneGlob ID: PAHS-007Z, Qiagen) according to the manufacturer’s protocol, and the reaction was run using a real-time PCR instrument. The PCR thermal cycling program was first denaturated at 95 °C for 10 min, followed by 40 cycles at 95 °C for 15 s and 60 °C for 1 min. The data analysis was performed using the RT^2^ Profiler PCR array data analysis online software (http://www.sabiosciences.com/pcrarraydataanalysis.php (Accessed 29 December 2024)). For the relative gene expression levels of fatty acid metabolism-related genes, the transcript levels of each gene were calculated relative to each other using raw cycle threshold values for each gene, normalized against ACTB, B2M, GAPDH, HPRT1, and RPLP0. The values shown are the log2-fold change relative to the average Ct value for all genes. The fold change values were calculated for each gene to determine if it was upregulated or downregulated for each treatment group vs. the control group. A fold change <1 was considered downregulation, and a fold change >2 was considered upregulation.

### 3.8. Quantitative Real-Time PCR (QPCR) of PPARα mRNA Expression

The total RNA was isolated using the RNeasy Mini kit (Qiagen) following the manufacturer’s instructions. The isolated RNA was then converted into cDNA using the SuperScript™ First-Strand Synthesis Kit (Invitrogen, Waltham, MA, USA). The expression of PPARα in the cancer cells was measured using real-time qPCR on the QuantStudio 6 Flex Real-Time PCR system (Applied Biosystems, Waltham, MA, USA) with the following cycle conditions: 95 °C for 5 min, followed by 40 cycles of 95 °C for 10 s and 60 °C for 30 s. GAPDH was used as the control. All primers were purchased from Macrogen, and the primer sequences are provided in Table 4. The 2^−∆∆CT^ method was used to determine the change in expression. All data were averaged from three independent experiments.

### 3.9. Molecular Docking of NSC3852 and PHA665752

The X-ray crystal structure of PPARα in complex with the antagonist GW6471 was retrieved from the Protein Databank [79] (PDB ID: 1KKQ) [24]. The structures of the compounds NSC3852 (CID: 19103) and PHA665752 (CID: 10461815) were retrieved from PubChem [80]. PyMol [81] version 2.5.2 was used to add the hydrogens, remove the water molecules, and save the protein chains and compound files in PDB format. The X-ray crystal structure has four ligand-binding domains (LBDs, chains A, B, C, and D), each containing a silencing mediator for the retinoid and thyroid hormone receptors (SMRT) co-repressor peptide and a GW6471 antagonist. Docking calculations were performed using each of the four chains to select the best one for further studies by considering how similar the predicted binding mode of GW6471 was to the pose determined in the X-ray crystal structure (PDB ID: 1KKQ) [24]. AutoDockTools version 1.5.7 [82] was used to add Kollman charges [83] to the protein and record the protein and compound files in PDBQT format before docking. AutoDock Vina version 1.1.2 [48,49] was used for the docking calculations. This software utilizes the Vina scoring function, which is derived by combining aspects of both knowledge-based potentials and empirical scoring functions [49]. The Vina scoring function includes terms for steric interactions, hydrophobic interactions, hydrogen bonding, and the number of active rotatable bonds between heavy atoms in the ligand for calculating the free energy of binding [49]. The co-crystallized antagonist was set as the center of a 30 Å-edge grid box. Ten docking poses per compound were generated using global searching exhaustiveness of 20 and an energy range cutoff of 5 kcal/mol. The root mean square deviation (RMSD) values between the docked pose of each GW6471 antagonist and its X-ray crystal structure pose were calculated using PyMol [81]. Docking into chain C gave a low RMSD value of 0.961 and showed favorable interactions between GW6471 and important residues. This finding is similar to other studies in which chain C was selected for docking calculations [47,84].

NSC3852 and PHA665752 were also docked into other known targets that affect the PPARα pathway, consequently leading to anti-tumor activities. Proteins with available X-ray crystal structures were used, and their structures in the inactive states were retrieved from the Protein Databank [79]. The same methods were used to prepare the proteins and the compounds and to perform the docking calculations. PyMol [81] was used to visualize the docked compounds and analyze the molecular interactions.

### 3.10. PPARα TR-FRET-Based Competitive Receptor Binding Assay

A TR-FRET-based competitive receptor binding assay was performed to determine ligand binding to PPAR alpha using Thermo Scientific Lanthascreen^TM^ competitive binding assay kits (catalog number PV4892) GW7647, GW6471, NSC3852, PHA665752, and DMSO.

The competitive binding affinity of GW7647, GW6471, NSC3852, and PHA665752 with PPAR alpha TR-FRET were performed as follows. In 96-well plates, 20 µL of 2X solution of GW7647, GW6471, NSC3852, PHA665752, and DMSO as the negative control were added. Then, 10 µL of 4X Fluormone^TM^ Pan-PPAR Green and 10 µL of 4X PPARα-LBD/Tb-anti-GST antibody were added to the 96-well plates, and the plates were gently mixed on an orbital plate shaker for 30 s. The assay plates were covered and incubated at room temperature for one hour. The absorbance was measured using SpectraMax Instruments (Molecular Devices, San Jose, CA, USA). Two wavelengths per well were taken with the instrument setting as follows: 340 nm excitation filter 30 nm bandwidth, 520 nm emission filter 25 nm bandwidth, 495 nm emission filter 10 nm bandwidth, delay time of 100 µs, and an integration time of 200 µs. The results were expressed as a ratio by dividing the emission signal at 520 nm by the emission signal at 495 nm.

## 4. Discussion

Peroxisome proliferator-activated receptors (PPARs) have recently been extensively studied. These receptors are categorized into three isotypes, namely PPARα, γ, and β/δ. Initially, they were believed to be essential metabolic regulators that controlled energy homeostasis in the body. However, in recent years, cancer has become a leading cause of human mortality globally, and as a result, the role of PPARs in cancer is increasingly being investigated. PPARs can either promote or suppress cancer, depending on various factors, including the type of receptor, cancer type, and stage [85,86]. Furthermore, the effectiveness of anti-cancer therapy based on drug-targeted PPARs also varies among the three receptors and the types of cancer [87].

Alteration of fatty acid metabolism in cancer has recently gained attention. The metabolic differences between normal and cancer cells have been considered a new strategy for tackling cancer, since dysregulated glycolysis and fatty acid metabolism have been linked with chemotherapy resistance in different types of cancers [88,89]. Cancer cells can modify their metabolism to produce ATP and essential macromolecules for cellular proliferation, division, and survival. The storage of excess fatty acids (FAs) in lipid droplets (LDs), which are cytoplasmic organelles in cells, is a significant implication of their uptake from outside the cell [90]. The accumulation of LDs in cancer cells serves not only to prevent toxicity and maintain lipid balance but also as a source of ATP and NADPH during cellular metabolic stress [90,91]. This occurs because the stored lipids are broken down through a process called β-oxidation, which produces acetyl-CoA through the oxidative degradation of FAs [92]. In this context, we explored how breast cancer cells modify their fatty acid metabolism, focusing on the importance of PPARα-targeted therapeutic approaches for fatty acid metabolism in breast cancer treatment.

Using the PPARE-Luciferase reporter, we confirmed that the PPARα pathway is active in our breast cancer cells, KAIMRC1 cells, without adding any exogenous ligand. Our results aligned with what has been reported in the literature—that these receptors are expressed in human breast cancer cell lines, and upon ligand binding, the activated receptor promotes cancer cell proliferation [93]. PPARs are nuclear transcription factors that bind with different types of ligands, including both physiological and pharmacological ones, and heterodimerize with retinoic acid X receptors. Physiological ligands that can bind with PPARα are mainly compounds derived from fatty acids and their derivatives [94]. Hence, the activation of PPARα in KAIMRC1 cells indicates that these cancer cells utilize an endogenous ligand to activate the receptor and initiate its signal.

Beta-oxidation is a metabolic pathway that helps cancer cells with essential supplies of acetyl-CoA, NADH, and FADH2 during aerobic glycolysis [95]. However, this process requires constant free fatty acids as substrates. To increase the pool of free fatty acids, cancer cells can use lipolysis of stored triglycerides, uptake of fatty acids from the environment, or lipogenesis. Previous studies have suggested that cancer cells activate lipid-scavenging pathways during nutrient deprivation, and they may expand the cellular fatty acid pool by consuming more exogenous fatty acids [91,96]. In metastatic tumors, fatty acid synthase is upregulated, and the expression of PPAR-α is highly amplified, which could upregulate the transcription of lipogenesis genes [95].

Activation of PPARα triggers the expression of crucial genes involved in fatty acid metabolism, increasing the production of reactive oxygen species. This, in turn, can promote the development of cancer [97]. Furthermore, the addition of leptin and glucose enhances breast cancer cell proliferation and upregulates PPARα, thereby indicating the involvement of PPARα in this process [98]. It is worth noting that cancer cells can modify their metabolism to produce ATP and essential macromolecules necessary for cellular proliferation, division, and survival. Alteration of the fatty acid metabolism in cancer has been linked with decreased apoptosis and drug resistance in cancer cells. Therefore, there is a high demand to identify new molecules targeting essential metabolic proteins to enhance the efficacy of anti-tumor drugs to combat cancer.

Fenofibrate is a PPAR-α agonist that has been used for several years to treat mixed dyslipidemia and hypertriglyceridemia. Fenofibrate causes a decrease in the usage of glucose by cancer cells, and instead, it prompts them to use fatty acids for their metabolic processes. Recent studies suggest that fenofibrate could have anti-tumor effects in different types of cancer, including breast cancer [99]. However, it has been reported that fenofibrate has an antiproliferative effect and induces apoptosis of triple-negative breast cancer cells independently from PPAR-α [99].

Upon a library screen, we identified two compounds (Figure 4) that can inhibit the PPARα pathway without binding to the receptor directly, as shown by molecular docking (Figure 8 and Figure 9). To confirm this result, we employed the TR-FRET assay to show that NSC3852 and PHA665752 do not bind PPARα, in contrast to the agonist GW7647 and antagonist GW6471 (Figure 7).

NSC3852 is a histone deacetylase inhibitor that induces apoptosis and differentiation in MCF-7 breast cancer cells upon the induction of oxidative stress [33,50]. Previous studies have reported that PHA665752 is a tyrosine kinase inhibitor that induces apoptosis combined with the mTOR inhibitor rapamycin. Moreover, it has been discovered that mice treated with PHA665752 have an antiangiogenic effect, which increases the angiogenesis inhibitor TSP-1 and decreases VEGF synthesis [100]. In breast cancer, silencing the EGFR gene in the MDA-MB-468 cell line, which is highly resistant to drugs, increases the cells’ sensitivity to PHA-665752. It has been reported that PHA-665752 works in synergy with erlotinib, an EGFR inhibitor, to decrease the viability of cancer cells.

PPARα promotes cancer growth and survival in multiple ways, such as increasing ketogenesis, fatty acid transportation, and synthesis [89]. Our study evaluated the impact of NSC3852 and PHA665752 on fatty acid metabolism in KAIMRC1 breast cancer cells. The gene expression studies showed that these two compounds had distinct expressions of the genes responsible for fatty acid metabolism. Notably, our analysis revealed that the expression of carnitine palmitoyltransferase 1 (CPT1C) was significantly decreased in the KAIMRC1 cells treated with PHA665752. CPT1C is a neuronal protein that regulates fatty acid oxidation and is crucial in regulating cellular energy homeostasis 68]. It is also considered a prognostic biomarker in several types of cancer. Studies have shown that CPT1C is highly expressed in cancer cells, and it favors tumor survival by helping cancer cells adapt to nutrient depletion and hypoxia via enhancing the oxidation of fatty acids and the production of ATP [77,95].

Interestingly, a previous study found that PPAR-α directly binds to the CPT1C promoter and activates its expression, suggesting that CPT1C is a downstream target gene of PPAR-α [101]. These findings on CPT1C can help to understand the metabolic activity of tumor cells and improve the design of better therapeutic strategies. Furthermore, our PCR analysis showed that protein kinase AMP-activated non-catalytic subunit gamma 3 (PRKAG3) was significantly downregulated in KAIMRC1 cells treated with PHA665762. PRKAG3 is essential in regulating cellular energy, but its expression and role in breast cancer cells remain unclear.

Growing evidence suggests that fatty acid-binding proteins (FABPs) are involved in cancer development. FABPs are small proteins that bind to lipids. There are 12 types of FABPs, each with a unique function in cancer development [102]. Increased levels of FABPs have been associated with cancer progression and metastasis [90]. Recent studies have shown that elevated levels of FABP4 are considered a prognostic marker in obesity and the development of breast cancer [103]. In our experiments, we found that decreased expression of genes involved in fatty acid oxidation, specifically FABP2 and FABP4, in the KAIMRC1 cells treated with PHA665762 resulted in reduced cell proliferation and increased apoptosis. However, in the cells treated with NSC3852, the expression of both genes was significantly upregulated compared to the cells treated with the positive control GW6471.

One of the primary requirements for the rapid proliferation of cancer cells is a high energy demand, which ketogenesis can provide. In our study, we observed that the ketogenesis rate-limiting enzyme 3-Hydroxymethylglutaryl-CoA synthase 2 (HMGCS2) expression decreased in the KAIMRC1 cells treated with PHA665762 but not in the cells treated with NSC3852. The expression of HMGCS2 is mainly regulated at the transcriptional level, and its promoter region contains a peroxisome proliferator response element (PPRE). When HMGCS2 binds to PPAR-α, it activates transcription and produces ketone bodies required for cellular energy [104]. Recent studies have shown that knocking down HMGCS2 in hepatocellular carcinoma cells inhibits cell proliferation [105]. In breast cancer cells, overexpression of HMGCS2 is considered an adverse prognostic factor [106]. Overexpression of HMGCS2 also increased the metastatic ability of breast cancer cells, MDA-MB-231, as reported by Martinez-Outschoorn and colleagues [107]. These results suggest that targeting HMGCS2 could be a potential therapeutic strategy for treating cancer cells.

Protein studies supported our gene expression analysis results. Our proteome profiler experiments revealed that the expression of apoptotic proteins, including catalase, FADD, and phospho-p53(S46), increased in the KAIMRC1 cells treated with PHA665752. The overexpression of catalase, a heme enzyme, indicates increased fatty acid oxidation, a marker for cells undergoing stress and death [108]. Furthermore, the Fas-associated Death Domain (FADD) is an adaptor protein that induces apoptosis. In cancer, loss of FADD inhibits apoptosis and induces tumor cell survival, making it a promising therapeutic option for treating cancer [70].

Additionally, the tumor suppressor protein p53 causes apoptosis in cells with DNA damage. Phosphorylation of p53 at serine 46 (S46) guides the cells to undergo apoptosis rather than cell cycle arrest [71]. Our results suggest that PHA665752 may activate the cell death of cancer cells by inhibiting the PPARα pathway independently rather than binding to PPARα directly.

On the other hand, NSC3852 may inhibit breast cancer cell proliferation by upregulating the expression of the apoptotic protein Claspin. Claspin is a human checkpoint protein with antiapoptotic activity that can be downregulated by RNAi and promote apoptosis [109].

Our results support PHA665752 and NSC3852 including KAIMRC1 cell death (Table 1) independently of binding the PPARα receptor, as shown in Figure 6 and Figure 7. To further strengthen our hypothesis that activation of the PPARα pathway by endogenous ligands leads to cancer cell proliferation and becomes dependent on this pathway, we compared the ability of PHA665752 and NSC3852 to kill cancer cells with different expression levels of PPARα. Based on The Human Protein Atlas [59], the cell with the lowest PPARα expression is the colorectal SW620 cell. First, we confirmed this information by checking the expression of PPARα in SW620 cells compared to KAIMRC1 cells using QPCR, as shown in Figure 8. Interestingly, when we tested the effects of PHA665752 and NSC3852 on the SW620 cells, the IC_50_ for killing the SW620 cell line was higher than KAIMRC1, as shown in Table 3, Figure 9. This data is very important to support the finding that cancer cells with a higher expression of PPARα may depend on this pathway to survive, and hence, blocking this pathway leads to its killing.

**Figure 13:**
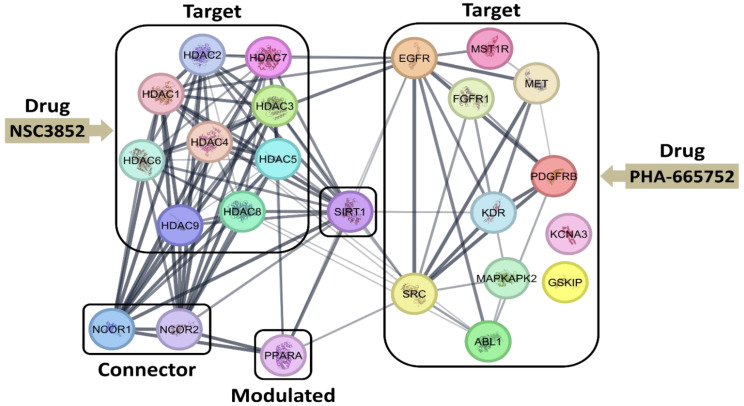
The protein–protein interaction network using the STRING app. Proteins are represented with color nodes, and interactions are represented with edges. Strong interactions are shown with thicker edges. The proteins are segregated into target, connector, and modulated proteins.

To understand the molecular mechanisms by which PHA665752 and NSC3852 block the PPAR alpha-activated pathway, we conducted a pathway analysis based on the molecular modeling of their targets (Table 2). As shown in Figure 13, this study revealed potential interactions among the network of proteins activated or deactivated due to compound treatment. Compound NSC3852, an HDAC inhibitor, inhibits certain HDACs, thereby affecting NCOR1 and NCOR2, ultimately modulating PPARα. Similarly, PHA665752 inhibits Met, which influences EGFR and SRC in modulating PPARα. Additionally, PHA665752 inhibits EGFR, impacting several HDACs and activating the HDAC-NCOR1 pathway to modulate PPARα. Interestingly, both compounds affect SIRT1, a potent modulator of PPARα. The STRING app, an open-source platform for complex network analysis, Cytoscape 3.10.2, was used to generate this figure. However, these pathways are just an example of many other pathways that could be involved. To understand further how these two compounds affect the PPAR alpha pathway and induce cell killing, a comprehensive study needs to be conducted, and, definitely, using artificial intelligence will help to dissect more precisely the involved mechanism.

## 5. Conclusions

In the present study, we discovered two novel compounds, PHA665752 and NSC3852, which can modulate the PPARα pathway independently rather than binding directly to the receptor itself, and we highlighted their impact on breast cancer cells. Both compounds regulate the PPARα pathway by controlling genes involved in the fatty acid oxidation process. Our findings suggest that these two compounds have opposing effects on fatty acid oxidation in the KAIMRC1 breast cancer cell line. To our knowledge, this is the first study reporting these two compounds as PPARα modulators. Although we do not fully understand their mechanism of action, our data provide new insights into the potential role of these compounds in targeting breast cancer cells. Our study lays the foundation for future research on PPARα-targeted therapeutics for fatty acid metabolism in breast cancer treatment.

## Figures and Tables

**Table 2:** Results of docking NSC3852, PHA665752, and co-crystallized inhibitors into different targets.

Target Name	PDB ID	Resolution (Å)	Co-Crystallized Inhibitor		Compound	
			Docking Score (kcal/mol)	RMSD	Docking Score (kcal/mol)	Interacting Residues
**Nicotinamide adenine dinucleotide-dependent protein deacetylase sirtuin-1 (SIRT1)**	**4I5I [51]**	2.50	−11.8	0.004	NSC3852	
					−7.6	Phe 273
Histone deacetylase 1 (HDAC1)	5ICN [52]	3.30	−6.1	2.492	NSC3852	
					−6.2	-
Histone deacetylase 2 (HDAC2)	8A0B [53]	1.75	−10.8	0.964	NSC3852	
					−6.9	Gly 143, His 145, and His 146
Histone deacetylase 4 (HDAC4)	6FYZ [54]	2.15	−10.9	0.932	NSC3852	
					−7.1	His 802 and His 803
Histone deacetylase 6 (HDAC6)	5EDU [55]	2.79	−8.4	1.340	NSC3852	
					−6.2	His 651 and Phe 680
Histone deacetylase 7 (HDAC7)	3ZNR [56]	2.40	−10.8	0.883	NSC3852	
					−7.0	Glu 840
Histone deacetylase 8 (HDAC8)	5FCW [57]	1.98	−9.7	0.870	NSC3852	
					−6.2	Tyr 154
Hepatocyte growth factor receptor (c-Met kinase)	7V3R [58]	1.70	−12.6	0.833	PHA665752	
					−9.2	Glu 1127

**Table 4:** Primer sequences for RT-PCR.

Gene Name	Sequence [5′ ⟶ 3′)
**PPARα Forward**	TTCGCAATCCATCGGCGAG
**PPARα Reverse**	CCACAGGATAAGTCACCGAGG
**GAPDH Forward**	ACCACAGTCCATGCCATCAC
**GAPDH Reverse**	TCCACCACCCTGTTGCTGTA

## Data Availability

Data will be made available on request.

## Abbreviations

Nuclear receptors (NRs), estrogen receptor (ER), retinoic acid receptor (RAR), retinoid x receptor (RXR), liver x receptors (LXRs) peroxisome proliferator receptors (PPARs), vitamin D receptor (VDR); abbreviations of all other nuclear receptors and their cofactors that are investigated in this study can be found through the following link: https://www.qiagen.com/fr/shop/pcr/primer-sets/rt2-profiler-pcr-arrays/?catno=PAHS-056Y#geneglobe (accessed on 12 December 2024), ligand-binding domains (LBD), Time-resolved fluorescence energy transfer (TR-FRET), half-maximal inhibitory concentration (IC50), Tumor growth factor (TGF), Vascular endothelial growth factor (VEGF), Epidermal growth factor receptor (EGFR), Fas-associated Death Domain (FADD), lipid droplets (LDs), King Abdullah International Medical Research Center Breast Cancer Cell (KAIMRC1), M.D. Anderson and MB stands for Metastasis Breast cancer cell (MDA-MB-231), Dukes C colorectal cancer cell (SW620).
