# Correction: Al Subait et al. Discovery of PPAR Alpha Lipid Pathway Modulators That Do Not Bind Directly to the Receptor as Potential Anti-Cancer Compounds. *Int. J. Mol. Sci.* 2025, *26*, 736

**DOI:** 10.3390/ijms26104654

**Published:** 2025-05-13

**Authors:** Arwa Al Subait, Raghad H. Alghamdi, Rizwan Ali, Amani Alsharidah, Sarah Huwaizi, Reem A. Alkhodier, Aljawharah Saud Almogren, Barrak A. Alzomia, Ahmed Alaskar, Mohamed Boudjelal

**Affiliations:** 1 Medical Research Core Facility and Platforms (MRCFP)-Drug Discovery Platform, King Abdullah International Medical Research Center (KAIMRC), King Saud Bin Abdulaziz University for Health Sciences, Ministry of National Guard Health Affairs (MNGHA), Riyadh 11481, Saudi Arabia; alsubaitar@kaimrc.edu.sa (A.A.S.); aliri@kaimrc.edu.sa (R.A.); huwaizis@kaimrc.edu.sa (S.H.); almogrena@kaimrc.edu.sa (A.S.A.); somaieb@mngha.med.sa (B.A.A.); askaras@mngha.med.sa (A.A.); 2 Clinical Laboratory Sciences Department, College of Applied Medical Sciences, King Saud Bin Abdulaziz University for Health Sciences, Ministry of National Guard Health Affairs (MNGHA), Riyadh 11481, Saudi Arabia; 3 King Abdulaziz and His Companions Foundation for Giftedness and Creativity (MAWHIBA), Riyadh 11481, Saudi Arabia; 445009532@pnu.edu.sa; 4 College of Science, King Saud University, Riyadh 11459, Saudi Arabia; 441203711@student.ksu.edu.sa; 5 Department of Pharmaceutical Sciences, College of Pharmacy, King Saud Bin Abdulaziz University for Health Sciences, Ministry of National Guard Health Affairs (MNGHA), Riyadh 11481, Saudi Arabia; khodierr@ksau-hs.edu.sa; 6 King Abdullah International Medical Research Center (KAIMRC), Ministry of National Guard Health Affairs (MNGHA), Riyadh 11481, Saudi Arabia

In the published publication [1], there was an error regarding the affiliation for Reem A. Alkhodier. In addition to affiliation 5, the updated affiliation should include: King Abdullah International Medical Research Center (KAIMRC), Ministry of National Guard Health Affairs (MNGHA), Riyadh 11481, Saudi Arabia. 

Ahmed Alaskar was misspelled in the original publication. The corrected spelling appears here. The authors state that the scientific conclusions are unaffected. This correction was approved by the Academic Editor. The original publication has also been updated.
